# Stimuli‐responsive delivery of therapeutics for diabetes treatment

**DOI:** 10.1002/btm2.10036

**Published:** 2016-10-03

**Authors:** Jicheng Yu, Yuqi Zhang, Hunter Bomba, Zhen Gu

**Affiliations:** ^1^ Joint Dept. of Biomedical Engineering University of North Carolina at Chapel Hill and North Carolina State University Raleigh NC 27695; ^2^ Center for Nanotechnology in Drug Delivery and Division of Molecular Pharmaceutics, UNC Eshelman School of Pharmacy University of North Carolina at Chapel Hill Chapel Hill NC 27599; ^3^ Dept. of Medicine University of North Carolina at Chapel Hill Chapel Hill NC 27599

**Keywords:** diabetes, drug delivery, insulin, stimuli‐responsive

## Abstract

Diabetic therapeutics, including insulin and glucagon‐like peptide 1 (GLP‐1), are essential for diabetic patients to regulate blood glucose levels. However, conventional treatments that are based on subcutaneous injections are often associated with poor glucose control and a lack of patient compliance. In this review, we focus on the different stimuli‐responsive systems to deliver therapeutics for diabetes treatment to improve patient comfort and prevent complications. Specifically, the pH‐responsive systems for oral drug delivery are introduced first. Then, the closed‐loop glucose‐responsive systems are summarized based on different glucose‐responsive moieties, including glucose oxidase, glucose binding protein, and phenylboronic acid. Finally, the on‐demand delivery systems activated by external remote triggers are also discussed. We conclude by discussing advantages and limitations of current strategies, as well as future opportunities and challenges in this area.

## 1 Introduction

Diabetes mellitus is a metabolic disorder that is characterized by high blood glucose (hyperglycemia).^1^, ^2^ Currently, diabetes affects over 415 million people worldwide, and that number is estimated to reach 642 million in 2040.^3^ In the United States, over 44.3 million people suffer from diabetes, which is about 12.9% of the population.^4^ Diabetes is due to either the body not producing enough insulin or cells not responding to the insulin that is produced.^1^, ^2^ There are three main forms of diabetes: type 1, type 2, and gestational diabetes. Type 1 diabetes, also known as insulin‐dependent or juvenile diabetes, results from a deficiency of insulin due to the T‐cell‐mediated destruction of its secretion source, *β*‐cells.^5^ Type 2 diabetes involves the inability of cells in the liver, muscles, and adipose tissue to respond to the normal functions of insulin.^6^, ^7^ Patients with type 1 diabetes are characterized by hyperglycemia and hypoinsulinemia, generally without insulin resistance. However, patients with type 2 diabetes are characterized by insulin resistance, which means their response to insulin is blunted, thus causing hyperglycemia.^6^ Gestational diabetes occurs during pregnancy due to the improper insulin responses, which usually disappears after the birth of the baby.^8^

**Figure 1:**
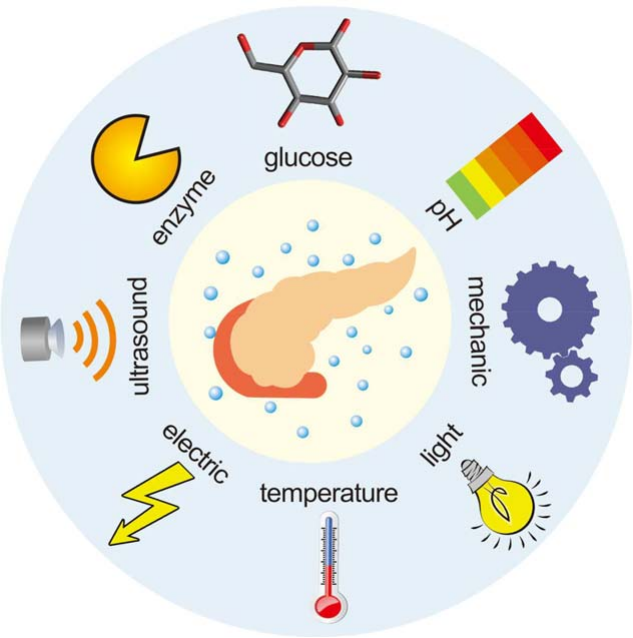
Schematic of a variety of stimuli to trigger delivery of diabetic therapeutics for diabetes treatment

The long‐term insulin deficiency in diabetic patients may lead to other severe complications such as retinopathy, neuropathy, cardiovascular disease, and ulceration.^9^ Persistent glycemic control is a key goal for diabetic patients. For patients with type 1 diabetes, insulin replacement is important to regulate blood glucose levels (BGLs) by mimicking natural fluctuations in insulin levels.^10^ For type 2 diabetes, exercise and regulation of meals can effectively delay disease progression.^7^ But for advanced type 2 diabetic patients, injection of insulin or other diabetic therapeutics, like glucagon‐like peptide 1 (GLP‐1), are also required to control the BGLs.^10^, ^11^ However, the traditional subcutaneous injection is painful and inconvenient. Moreover, this open‐loop administration is often associated with inadequate glucose control.^10^ In recent decades, a number of technologies have been developed to overcome the limitations and drawbacks of such injection therapy. Among them, strategies utilizing stimuli‐responsive delivery systems are highly desirable to regulate glycemia with minimal patient effort and improve the quality of life for diabetic patients.^12^, ^13^ In this review, we will focus on the different stimuli‐responsive systems for diabetic therapeutic delivery, including pH‐responsive, glucose‐responsive, and external physical trigger‐activated systems (Figure 1). The future opportunities and challenges will also be discussed.

## 2 pH‐responsive systems for oral drug delivery

Oral delivery is considered to be the most patient‐friendly method for insulin administration.^14^, ^15^, ^16^, ^17^ However, the low pH of gastric medium in the stomach and various digestive enzymes in the gastrointestinal tract may degrade insulin, leading to low efficacy of therapy.^18^, ^19^ To enhance the bioavailability and treatment efficacy, numerous pH‐responsive based systems have been exploited, such as enteric capsules and particles,^20^ which are able to protect protein drugs from the harsh gastric environment, as well as allow specific release in the intestine.^21^, ^22^, ^23^ These drug carriers are stable under acidic conditions in the stomach, but can rapidly release cargoes under neutral pH in the intestine. In the following section, we will introduce the recent pH‐responsive oral delivery systems based on polymeric and inorganic materials.

**Figure 2:**
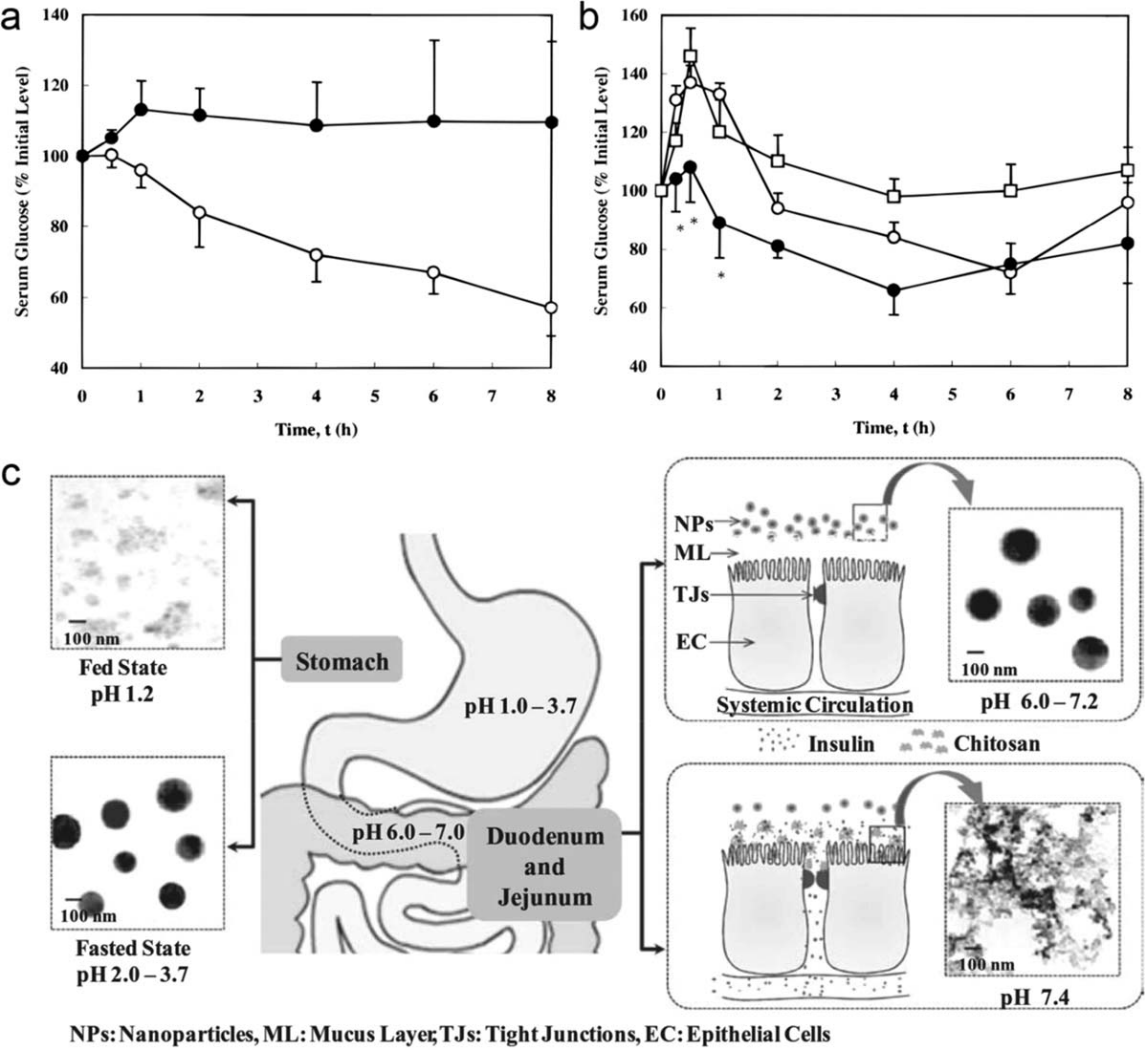
(a) Blood glucose levels in diabetic rats following oral administration of 25 IU/kg body weight doses contained in (○) P(MAA‐g‐EG) microspheres and (•) insulin solutions. (b) Blood glucose response in healthy rats following the oral administration of P(MAA‐g‐EG) microspheres containing insulin doses of (○) 25 IU/kg body weight and (•) 50 IU/kg body weight and (□) insulin solutions (50 IU/kg body weight). (c) Schematic illustrations of the presumed mechanism of the paracellular transport of insulin released from pH‐responsive NPs. Reproduced with permission from Refs. 22, 34

Peppas and coworkers pioneered the utilization of pH‐responsive complexation gel for oral delivery of insulin.^22^, ^24^, ^25^, ^26^ They loaded insulin into poly(methacrylic‐*g*‐ethylene glycol) (P(MAA‐*g*‐EG)) hydrogels, and orally administered these polymeric microspheres to streptozotocin‐induced type 1 diabetic rats.^22^ The hydrogels were prevented from swelling under the acidic condition in the stomach because of the formation of intermolecular polymer complexes by hydrogen bonding between the carboxylic acid protons and the etheric groups on the grafted chains, which efficiently protected insulin form degradation. In the neutral environment of the intestine, the intermolecular polymer complexes disassociated and the gel swelled, resulting in a rapid insulin release (Figure 2a, b). They demonstrated that the insulin was sufficiently absorbed in the upper small intestine with an obvious hypoglycemic action; the bioavailability was 4.6–7.2% in a diabetic rat model.^24^ They further conjugated insulin with transferrin to enhance the bioavailability of oral insulin.^25^ Transferrin can be uptaken by the epithelial cells to increase the permeability of insulin across the epithelial barrier, and it can also prevent insulin degradation by intestinal enzymes. Integrating the transferrin with the pH‐responsive hydrogels resulted in a 22‐fold net increase in insulin permeability. In another work, they modified P(MAA‐*g*‐EG) with wheat germ agglutinin (WGA) to improve mucoadhesive ability.^26^ WGA is a class of lectins, which can bind to the luminal surface of the small intestine.^27^ In addition, WGA showed minimal binding to mucin at a low pH in the stomach. They revealed this system could significantly improve the overall adhesion to a cellular monolayer in an in vitro study.

In addition to synthetic polymers, natural polymers such as chitosan (CS) also show a potential role in pH‐responsive oral insulin delivery.^23^, ^28^, ^29^, ^30^ CS is a natural cationic polysaccharide, which exhibits good mucoadhesiveness and the capability to open tight junctions.^28^ More importantly, its physicochemical properties also depend on the surrounding pH value. Sung and coworkers developed a pH‐responsive NP consisting of CS and poly(*γ*‐glutamic acid) (*γ*‐PGA) for oral insulin delivery.^31^ Since both CS and *γ*‐PGA were ionized at pH 2.5–6.6, the prepared NPs were stable by the electrostatic interaction between CS and *γ*‐PGA. In contrast, at pH 7.4, CS was deprotonated, which led to the collapse of NPs and subsequent insulin release. Furthermore, the positive charged CS shell could increase the paracellular permeability by transiently opening the tight junctions. After oral administration in diabetic rats, these NPs could effectively decrease the BGLs. To improve the stability of the NPs in a broader pH range, magnesium sulfate (MgSO_4_) and tripolyphosphate sodium (TPP) were further introduced to construct a multi‐ion‐crosslinked NPs^32^, ^33^, ^34^ (Figure 2c). The introduction of MgSO_4_ and TPP also significantly increased their loading efficiency and content of insulin. The in vivo results demonstrated a significant hypoglycemic effect in diabetic rats by oral administration, and the corresponding relative bioavailability of insulin was about 15%. Through conjugating thiol groups on the CS chain, its mucoadhesive capability can be enhanced due to the disulfide formation between thiolated CS and the cysteine residues of the mucin.^35^ Yin et al. synthesized trimethyl chitosan‐cysteine conjugate and formed NPs with insulin through the electrostatic interaction.^36^ These polyelectrolyte NPs exhibited a significant enhanced mucoadhesion and permeability in rat intestine, which allowed a better hypoglycemic effect.

Enteric capsules or enteric coating are also utilized to improve the drug efficiency by protecting insulin from the digestive enzymes in the stomach. Mitragotri and coworkers loaded mucoadhesive intestinal patches in an enteric capsule for oral insulin delivery.^37^ The capsule could protect insulin‐loaded patches in the acidic environment of the stomach, while release them in the intestine. The released patches adhered to the intestinal mucosal layer to promote insulin absorption. Recently, they further loaded the intestinal devices with a permeation enhancer into a capsule coated with a pH‐responsive enteric coating to improve oral absorption of insulin.^38^ Sung and coworkers also filled the freeze‐dried insulin‐loaded NPs into an enteric‐coated capsule.^39^ The Eudragit^®^ S100 or Eudragit^®^ L100‐55‐coated capsules remained intact in the acidic environment of the stomach, while rapidly dissolved and released NPs in the neutral environment of the intestine. In vivo studies in diabetic rats indicated that relative bioavailability of insulin was approximately 20%.

Apart from polymeric materials, inorganic NPs have also been explored as insulin carriers due to their high loading capability and good compatibility with insulin. Sun et al. utilized mesoporous silica NPs (MSN) to increase the loading capacity of insulin.^40^ These inorganic NPs were further coated with pH‐sensitive dextran‐maleic acid (Dex‐MA) and then grafted with glucose‐sensitive 3‐amidophenylboronic acid (APBA). The Dex‐MA shell could shrink and block the pores to inhibit insulin release in the acidic pH of the stomach, but swell to allow insulin diffuse at neutral pH in the intestine. Verma et al. designed a vitamin B12 functionalized layer by layer calcium phosphate NP to improve oral delivery of insulin.^41^ Vitamin B12 was chosen due to its high paracellular and receptor mediated uptake efficiency in the intestine, as well as its low pKa (∼1.8) that changed the surface charge of NPs as a function of pH.

Shrestha et al. developed multistage pH‐responsive mucoadhesive nanocarriers to controllably deliver GLP‐1 and the enzyme inhibitor, dipeptidyl peptidase‐4 (DPP4) inhibitor.^42^ GLP‐1 is a neuropeptide secreted by intestinal L cells to stimulate insulin secretion and suppress glucagon secretion in a glucose‐dependent manner.^43^ Moreover, GLP‐1 no longer stimulates insulin under normoglycemia, which can avoid hypoglycemia.^43^ However, the half‐life time of GLP‐1 is relatively short (1–2 min), since it is quickly degraded by the enzyme DDP4.^43^, ^44^ Thus, the DPP4 inhibitor was co‐delivered with GLP‐1 to enhance the therapy efficiency in this system. GLP‐1 was loaded into the pores of CS‐coated undecylenic acid modified thermally hydrocarbonized porous silicon (UnPSi) NPs, and then hydroxypropylmethyl cellulose acetate succinate (HPMCAS‐MF), an enteric polymer, further formed a pH‐responsive polymeric matrix to encapsulate the DPP4 inhibitor and GLP‐1 loaded NPs. Since HPMCAS‐MF is only soluble at pH ≥ 6, the HPMCAS‐MF coating could effectively prevent the gastric degradation of GLP‐1. When under a neutral pH of intestine, the chitosan‐modified PSi NPs were released and showed augmented intestinal permeability in an in vitro cell‐based intestinal epithelium model.

## 3 Glucose‐responsive drug delivery

Closed‐loop based smart insulin delivery that can mimic the pancreas' *β*‐cells to secrete insulin in response to hyperglycemia has gained an increasing amount of attention.^12^, ^13^, ^45^, ^46^ Usually, such closed‐loop delivery systems are composed of a glucose monitoring module and a glucose‐triggered insulin releasing module.^12^, ^45^ One notable example is the electronic/mechanical insulin pump, which consists of a continuous glucose sensor and an external insulin infusion pump.^47^, ^48^ The insulin infusion rate can be adjusted in response to the BGL signal from the glucose sensor in these wireless, portable, and wearable systems.^49^, ^50^ Conversely, synthetic smart insulin delivery systems have been heavily explored to achieve closed‐loop delivery from the use of material design and formulation engineering.^12^, ^13^, ^45^, ^46^ Typically, these chemical closed‐loop systems, which based on glucose‐responsive materials, can sense an increase in BGLs and respond accordingly to release a certain amount of insulin for glycemia regulation.^12^, ^45^ Glucose oxidase (GOx), glucose binding proteins (GBPs), and phenylboronic acid (PBA) are the three most used glucose‐sensing moieties.^45^, ^46^ We will introduce the mechanism of these diverse systems, respectively, and discuss the most recent advances in this section.

### 3.1 GOx‐based systems

GOx is the most prevalent among the various glucose‐responsive moieties described in the literature.^13^ GOx presents a high level of specificity for glucose, and it converts glucose into gluconic acid in a biological environment:

$$\text{Glucose}+O_{2}+H_{2}O\;\overset{GOx}{\to }\text{gluconic}\;\text{acid}+H_{2}O_{2}.$$

Accompanying the oxidation of glucose, a local acidic, hypoxic, and high H_2_O_2_ concentration environment will be generated.^51^ Based on these changes, several stimuli‐sensitive materials have been developed to achieve glucose‐responsive action.^52^, ^53^, ^54^, ^55^

With the generation of gluconic acid, local pH decreases rapidly, which can serve as the trigger of insulin release.^56^, ^57^, ^58^, ^59^ For example, Ishihara and coworkers developed a pH‐sensitive poly(amine) membrane for glucose‐responsive delivery.^60^ In the presence of glucose, the protonation of tertiary amino groups under acidic conditions increased the water content of this poly(amine) membrane, further enhancing the permeability of insulin. Langer and coworkers designed a GOx‐immobilized polymeric system to achieve the controlled release of insulin.^61^ The reduced pH due to the enzymatic reaction of glucose increased the solubility of trilysyl insulin dramatically, leading to a higher drug diffusion rate in response to glucose concentration. Peppas and coworkers utilized a pH‐sensitive hydrogel for glucose‐responsive insulin delivery.^62^, ^63^ Through the swelling/collapsing process of the hydrogel in response to the changes in pH, the release rate of insulin could be controlled in a pulsatile manner.

With the development of acid‐sensitive materials and nanotechnology, a variety of new formulations and relative designs have been exploited. Gordijo et al. designed a glucose‐responsive bioinorganic nanohybrid membrane for closed‐loop insulin delivery.^64^ The bioinorganic membrane consisted of nanohybrids of bovine serum albumin (BSA)‐MnO_2_‐enzymes (GOx and catalase) integrated with pH‐sensitive hydrogel nanoparticles (NPs). Since the rapid oxygen consumption and the inactivation of GOx caused by hydrogen peroxide may slow down the glucose oxidation, the enzyme catalase (CAT), was introduced into the system, which has been widely incorporated with GOx in various systems as an H_2_O_2_ scavenger. CAT can convert H_2_O_2_ to water and O_2_ to quench undesired H_2_O_2_ and enhance the activity of GOx. Meanwhile, inorganic MnO_2_ NPs can also catalyze the conversion of H_2_O_2_ to stable GOx. The mechanical properties of crosslinked BSA were further improved due to the introduction of MnO_2_ NPs. Under a high glucose concentration, a decrease in pH generated by glucose oxidation caused the pH‐sensitive hydrogel NPs to shrink, leading to a fourfold increase in insulin release. In addition, the same group utilized this bioinorganic nanocomposite membrane as a glucose‐responsive plug to regulate insulin release from a reservoir made of medical grade silicone.^65^ In vitro experiments showed that the insulin release profile exhibited a typical pulsatile pattern when changing glucose concentration between normal and hyperglycemic levels for several cycles. Through intraperitoneal implantation, the BGLs of diabetic rats could be controlled in the normal range for 4 days. Furthermore, the glucose‐responsiveness of the device was also demonstrated in diabetic rats by a glucose challenging. The BGLs return from a hyperglycemic state to the normal level within 60 min.

Anderson's group also reported a pH‐sensitive sponge‐like matrix as an insulin reservoir.^52^ The glucose‐responsive microgels were prepared by crosslinking chitosan using TPP, a medical biocompatible polymer, insulin, and glucose‐specific enzymes (GOx and CAT), which were entrapped in this non‐covalent crosslinked polymeric matrix. To improve stability and immunogenicity of enzymes, both GOx and CAT were encapsulated into the nanocapsules. When subjected to hyperglycemic conditions, hydrogen ions were generated during glucose oxidation by GOx protonated chitosan and the microgel system swelled more than fivefold in volume, subsequently enhancing insulin release from the swelling sponge.

Besides pH‐induced volume change, acid hydrolysis is also applied in GOx‐mediated glucose‐responsive systems. Langer, Anderson, and coworkers developed an injectable and acid‐degradable polymeric network for self‐regulated insulin delivery.^53^ Insulin, GOx, and CAT were entrapped in an acid‐sensitive nanoparticle made of acetal modified dextran (*m*‐dextran) through a double emulsion‐based solvent evaporation/extraction method. In this system, *m*‐dextran was hydrolyzed into water‐soluble dextran in the presence of produced gluconic acid, and caused the dissociation of the NPs, leading to subsequent insulin release. To make the formulation injectable and overcome burst release, the NPs were coated with oppositely charged polymers, chitosan, and alginate, respectively, and mixed to form a nanocomposite‐based porous network. The gel‐like nano‐network effectively dissociated to release insulin at a hyperglycemic level (400 mg/dL), but showed insignificant release at a normal level (100 mg/dL). After a single subcutaneous injection into type 1 diabetic mice, the nano‐network was demonstrated to provide improved glycemia regulation for up to 10 days.

Using a similar response mechanism, Gu and coworkers synthesized a pH‐sensitive diblock copolymer, PEG‐Poly(Ser‐Ketal), and prepared the polymersome‐based nanovesicles to encapsulate insulin and glucose‐specific enzymes.^66^ Glucose was easy to passively transport across the bilayer membrane of the polymersome, and converted into gluconic acid by GOx, which was further hydrolyzed amphiphilic PEG‐Poly(Ser‐Ketal) to hydrophilic PEG‐Polyserine. The switch of water‐solubility resulted in the collapse of the nanovesicles and the subsequent release of insulin in a glucose‐mediated process. After being mixed with a thermoresponsive and biodegradable polymer (PF127), the suspension containing the vesicles was subcutaneously injected into the back of mice and quickly formed a stable hydrogel. In vivo performance of the hydrogel showed that the glucose‐responsive nanovesicles were able to maintain the normal BGL for up to 5 days.

**Figure 3:**
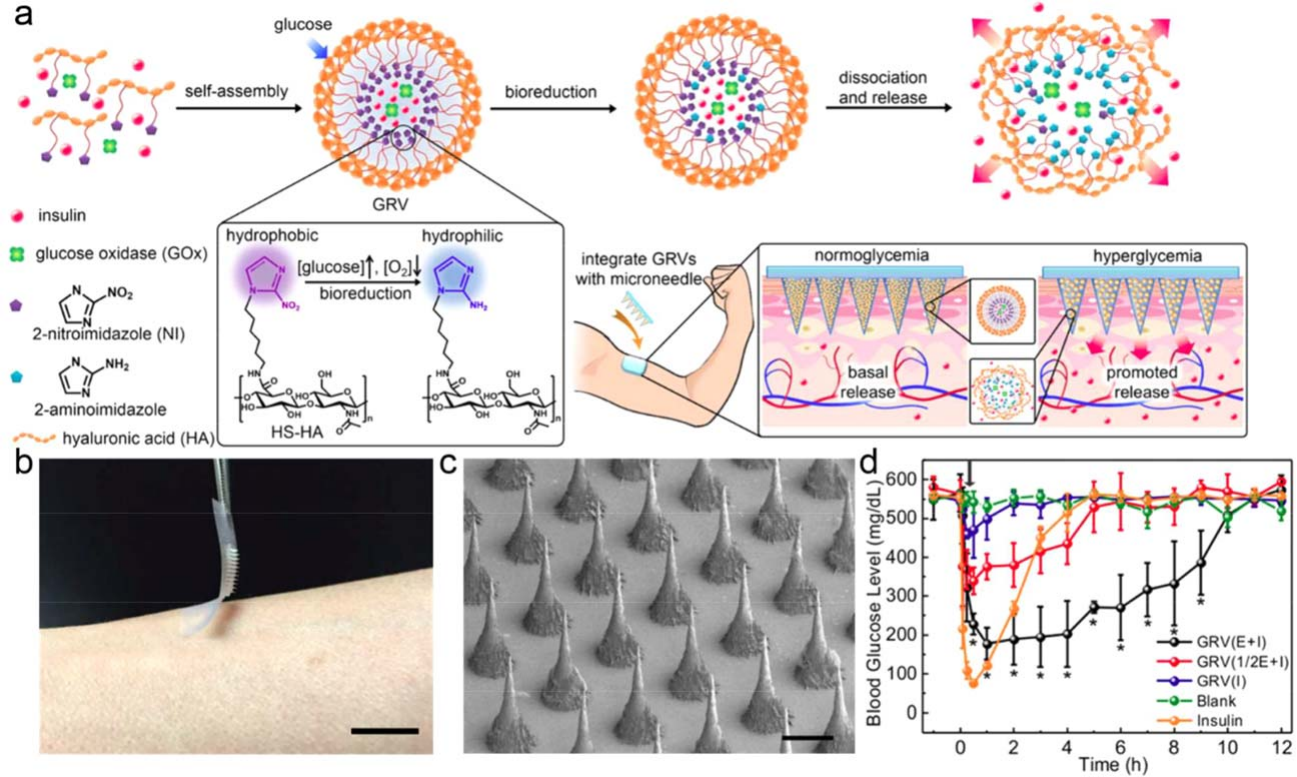
Hypoxia‐sensitive vesicle loaded MN‐array patches for glucose‐responsive insulin delivery. (a) Schematic of the formation and release mechanism of GRV and the GRV‐containing MN‐array patch for in vivo insulin delivery triggered by a hyperglycemic state. (b) Photograph of the MN‐array patch. (Scale bar: 1 cm) (c) SEM image of MN‐arrays. (Scale bar: 200 μm) (d) BGLs in diabetic mice after treatment with blank MNs containing only cross‐linked HA, MNs loaded with human recombinant insulin, MNs loaded with GRV(E + I), MNs loaded with GRV(1/2E + I), or MNs loaded with GRV(I). Reproduced with permission from Ref. 54

Recently, Yu et al. applied a local hypoxic environment generated by rapid oxygen consumption during enzymatic glucose oxidation as a trigger to achieve fast glucose responsiveness.^54^ They conjugated a hydrophobic hypoxia‐sensitive molecule, 2‐nitroimidazole (NI), onto the side chains of hyaluronic acid (HA). NI can be bioreduced to hydrophilic 2‐aminoidiazoles by a series of nitroreductases coupled to bioreducing agents like NADPH, a plentiful coenzyme in tissues under hypoxic conditions.^67^, ^68^ Since the synthesized hypoxia‐sensitive HA is amphiphilic, it can readily form a nanoscale glucose‐responsive vesicle (GRV) in aqueous solution, entrapping both insulin and GOx inside (Figure 3a). Under a hyperglycemic state (400 mg/dL), the dissolved oxygen can be quickly consumed due to the glucose oxidation by GOx, leading to a local hypoxic condition. Thereafter, hypoxia‐sensitive HA was bioreduced to a water‐soluble product, resulting in the dissociation of GRVs and subsequent insulin release. In vitro results revealed that the GRVs with a novel trigger mechanism presented a fast reversible control of insulin release between a normal and hyperglycemic state. To achieve ease of administration, GRVs were loaded in to a microneedle (MN)‐array patch for painless and continuous insulin delivery^69^, ^70^ (Figure 3b, c). Under a normal BGL, the GRVs were stable in the MN; however, they quickly disassembled to release insulin when exposed to high interstitial fluid glucose in vascular and lymph capillary networks. In vivo experiments displayed that one patch could decrease BGL to a normoglycemic state within 30 min and could be maintained for up to 4 hr without any risk of hypoglycemia in a type 1 diabetic mouse model (Figure 3d). This fast glucose‐responsiveness can be attributed to the fast generation of the local hypoxic microenvironment. In addition, the relatively low diffusion rate of oxygen may also promote the activation of GRVs.^71^, ^72^

Utilizing this hypoxia‐sensitive material, Ye et al. designed a synthetic glucose‐signal amplifier (GSA) for smart insulin delivery.^73^ The GSAs encapsulating GOx, *α*‐amylase (AM), and glucoamylase (GA), as well as *α*‐amylose were loaded in the MNs. Once subjected to the elevated BGLs, the GSA rapidly disassociated to release the encapsulated enzymes, which converted *α*‐amylose into glucose. The amplified glucose signal further simulated the externally positioned *β*‐cell capsules to secrete insulin. They demonstrated that the system consisting of ∼10^7^ *β*‐cells could decrease BGLs and maintain them at a reduced level for up to 10 hr in a diabetic mouse model.

### 3.2 Glucose‐binding protein‐based systems

Another non‐enzymatic strategy to achieving glucose‐triggered insulin delivery is based on the reversible interaction between glucose and glucose binding moieties. GBPs, such as lectins, are a group of natural carbohydrate‐binding proteins.^74^, ^75^ One of the most commonly used lectin for glucose‐responsive insulin delivery is concanavalin A (Con A), which is derived from the jack bean.^76^ Con A is formed with two dimers and has four binding sites for D‐glucose, D‐mannose, and polysaccharides.^77^, ^78^, ^79^ Kim and coworkers utilized gluconic acid‐modified insulin (G‐Ins) to complementarily bind to Con A to achieve a glucose‐induced release of insulin.^79^, ^80^, ^81^ When exposed to glucose solution, insulin can be released through competitive binding.

An alternative glucose regulated insulin delivery matrix is based on the affinity between Con A and natural polysaccharide polymers.^82^, ^83^, ^84^ Ying and coworkers utilized Con A as a crosslinker to form a dextran‐based NP.^85^, ^86^ Once exposed to an elevated glucose concentration, these NPs quickly dissolved due to the competition binding of glucose to Con A, leading to the release of encapsulated insulin. Nakamae and coworkers developed a glucose‐responsive hydrogel consisting of Con A and poly(2‐glucosyloxyethyl methacrylate) (poly(GEMA)).^87^ The introduction of Con A increased crosslinking density due to the complexation between Con A and poly(GEMA). In the presence of glucose, the dissociation of the poly(GEMA)‐Con A complex facilitated the swelling of hydrogel, and the swelling ratio was dependent on the glucose concentration. Yin et al. also designed several glucose‐responsive microgels by the reversed‐phase emulsion crosslinking method.^88^, ^89^ Natural polysaccharide polymers, such as dextran and chitosan, were chosen to form the complexes with Con A. In vitro experiments demonstrated that the microgels swelled or hydrolyzed when glucose concentration increased, thus promoting the release of insulin.

Ye et al. reported a nanogel made of Con A interpenetrated in a chemically crosslinked network of poly(*N*‐isopropylacrylamide) (poly(NIPAM)).^90^ The semi‐interpenetrating‐structured nanogels can swell and become stable in less than 1 s after adding glucose over a concentration range of 50 μM to 20.0 mM at a physiological pH of 7.4. A pulsatile profile of insulin release can be modulated in response to glucose concentration in vitro. A Con A‐gated MSN nanocontainer was developed for glucose‐responsive delivery by Wu et al.^91^ In their system, the external surfaces of MSNs with cylindrical channels were modified with mannose epitopes, and the subsequent capping with Con A entrapped the cargo within the pores. The Con A‐gated pores could be re‐opened by addition of glucose due to the competitive binding. The release rate correlated well with the glucose concentration.

### 3.3 PBA‐based systems

Besides natural glucose‐binding molecules, synthetic molecules have also been used in diabetes diagnosis, glucose sensors, and glucose‐responsive insulin delivery.^92^, ^93^ PBA is a synthetic molecule that is able to reversibly bind to 1,2‐ or 1,3‐*cis*‐diols, including many kinds of sugar, to form cyclic esters.^92^, ^94^, ^95^ Since the first discovery of the reversible interaction between PBA and sugar in 1959,^96^ PBA‐based glucose‐responsive insulin delivery has been widely exploited for closed‐loop insulin delivery.^97^, ^98^, ^99^, ^100^, ^101^ However, the lack of selectivity toward glucose limits the in vivo application of PBA‐based systems.

Kataoka, Okano and coworkers developed a glucose‐responsive insulin‐releasing polymer device based on the reversible binding between PBA groups and diol‐containing molecules.^102^ In their system, they applied a gel bead containing PBA groups as the drug reservoir, and gluconic acid‐modified insulin (G‐Ins) was bound onto PBA gel beads. The release rate of G‐Ins could be tuned in response to varying glucose concentration. Zhao et al. exploited a MSN‐based glucose‐responsive drug delivery system for controlled release of insulin and cyclic adenosine monophosphate (cAMP).^103^ cAMP can activate Ca^2+^ channels of pancreas *β*‐cells and thus stimulate insulin secretion. The exterior surface of MSNs was decorated with PBA, and then gluconic acid‐modified insulin (G‐Ins) could bind to PBA as a gatekeeper to encapsulate cAMP in the mesopores through a PBA‐diol interaction. Once exposed to glucose, the capped G‐Ins were replaced by free glucose molecules, and the loaded cAMP was simultaneously released to achieve the regulation of glycaemia.

Glucose‐responsive systems can also be achieved by complexation between PBA‐containing polymer and polyol polymers like polyvinyl alcohol (PVA) and glycopolymers.^104^ Ma et al. utilized the reversible interaction between PBA and glycosyl to control insulin release from a PBA‐based complex micelle.^105^ The glucose‐responsive micelles were prepared by self‐assembly of a PBA‐containing block copolymer poly(ethylene glycol)‐*block*‐poly(acrylic acid‐*co*‐acrylamidophenylboronic acid) (PEG‐*b*‐P(AA‐*co*‐APBA)) and a glycopolymer poly(acrylic acid‐*co*‐acrylglucosamine) (P(AA‐*co*‐AGA)). The micelles with a PEG shell were stable under physiological condition, but would undergo dissociation in the presence of glucose due to the competitive exchange between free glucose and glycosyl. Similarly, a glucose‐responsive complex micelle formed by the complexation between a PBA‐containing block copolymer poly(ethylene glycol)‐*b*‐poly(aspartic acid‐*co*‐aspartamidophenylboronic acid) (PEG‐*b*‐P(Asp‐*co*‐AspPBA)) and a glycopolymer poly(aspartic acid‐*co*‐aspartglucosamine) (P(Asp‐*co*‐AGA)) was demonstrated to promote insulin release under hyperglycemic conditions by Yang et al.^106^

Instead of utilizing two synthetic polymers composed of PBA and diol, respectively, Li and coworkers designed an amphiphilic block glycopolymer poly(D‐gluconamidoethyl methacrylate‐*block*−3‐acrylamidophenylboronic acid) (p(AAPBA‐*b*‐GAMA)) based on PBA and a carbohydrate.^107^ Through crosslinking inter‐ and intramolecular complexation between the diols of the carbohydrates and PBA, the glycopolymers self‐assembled into nanoparticles. The introduction of free glucose would competitively bind to the PBA domain and simultaneously break down the crosslinking interaction between AAPBA and GAMA blocks, leading to a glucose concentration‐dependent insulin release. Wu et al. prepared mono‐disperse injectable nanogels with poly(NIPAM), poly(3‐acrylamidophenylboronic acid) using maleic acid–dextran as a crosslinker.^108^ The biocompatible dextran can take part in the glucose‐responsive function of the nanogels through the interaction between the numerous diols groups of dextran and PBA. In vitro studies showed that addition of glucose caused nanogel swelling up to twofold of the original size, and this volume change was reversible by removing glucose. The encapsulated insulin can rapidly diffuse from the nanogel when nanogels swelled in the presence of glucose.

**Figure 4:**
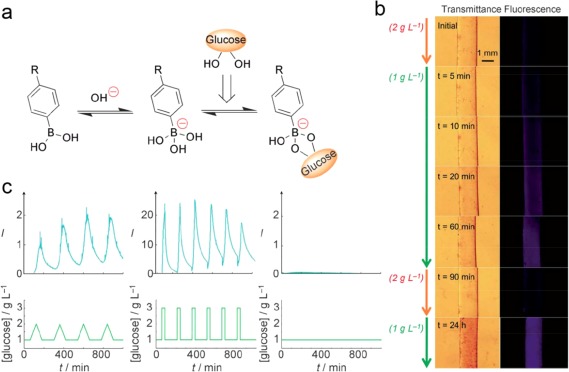
Gels composed of AmECFPBA for self‐regulated insulin delivery. (a) Glucose‐dependent equilibrium of phenylboronic acid. (b) Time‐course (left) transmittance and (right) 8‐anilino‐1‐naphthalene sulfonic acid (ANS) fluorescence top‐view images of a cylinder‐shaped piece of gel when changing the glucose concentration under pH 7.4 and 37°C. (c) (Top) Time‐course changes in the fluorescence intensity of FITC‐labeled bovine insulin released from the gel under physiological conditions (pH 7.4, I = 0.15, 37°C). (Bottom) Temporal patterns of the fluctuation in glucose concentration, investigated in each experiment. Reproduced with permission from Ref. 110

Besides competitive binding with PBA by free glucose, an alternative approach to achieve glucose responsiveness is to utilize the charge change of PBA when interacting with glucose.^45^ Kim and coworkers synthesized an amphiphilic copolymer poly(ethylene glycol)‐*b*‐poly(styreneboroxole) (PEG‐*b*‐PBOx) to prepare a polymersome‐based vesicle to encapsulate water‐soluble insulin.^109^ In the presence of monosaccharides, the hydrophobic PBO block interacted with glucose and transformed into negatively charged phenylboronate, which led to the disassembly of polymersome. A self‐regulated insulin release profile was displayed in response to the concentrations of monosaccharides. Matsumoto et al. synthesized a new PBA derivative, 4‐(2‐acrylamidoethylcarbamoyl)−3‐fluorophenylboronic acid (AmECFPBA) possessing *para*‐carbamoyl and *meta*‐fluoro substituents.^110^ The introduction of strong electron‐withdrawing substituents decreased the pKa value of this new derivative to 7.2, a value appreciably lower than physiological pH (7.4), thus suggesting glucose sensitivity under such a condition. Through conjugation of AmECFPBA to a thermo‐sensitive PNIPMAAm polymer, they obtained a glucose‐responsive hydrogel under physiological pH and temperature (pH 7.4 and 37°C). The gel was completely dehydrated under a normoglycemic condition, whereas it became remarkably hydrated when the glucose concentration increased (Figure 4a). The threshold value of the volume change was in agreement with that of normoglycemia (100 mg/dL) under physiological conditions when the fraction of AmECFPBA was 0.075 molar. With the decrease of glucose concentration, the gel can promptly form a hydrophobic skin layer through surface dehydration, thus inhibiting both further glucose penetration and loss of contents (Figure 4b). Through a surface‐limited mechanism, a continuous control of the insulin release rate could be achieved by altering glucose concentrations between 100 mg/dL and 200 mg/dL (Figure 4c). Notably, this non‐equilibrium mechanism significantly shortened the response time due to the short diffusion distance across the skin layer, allowing the insulin release rate close correspond with the external glucose levels.

Integrating with a silver (Ag) NP core, Wu et al. developed a multifunctional hydrid nanogel for simultaneous optical glucose sensing and self‐regulated insulin release.^111^ The Ag nanoparticle cores were covered by a copolymer gel shell of poly(4‐vinylphenylboronic acid‐*co*−2‐(dimethylamino)ethyl acrylate) (p(VPBA‐DMAEA)), which underwent swelling or shrinking in response to the glucose concentration. The corresponding volume change could convert the glucose concentration into a fluorescent signal through the Ag NPs, and simultaneously tuned the insulin release rate in a glucose‐dependent manner.

## 4 On‐demand insulin delivery based on external physical triggers

Apart from applying physiological factors to trigger insulin release, many efforts have been devoted to creating an on‐demand anti‐diabetic drug release system with a short response time through the use of external remote triggers including ultrasound, light, and temperature signals.^112^, ^113^, ^114^

### 4.1 Ultrasound‐triggered drug delivery

**Figure 5:**
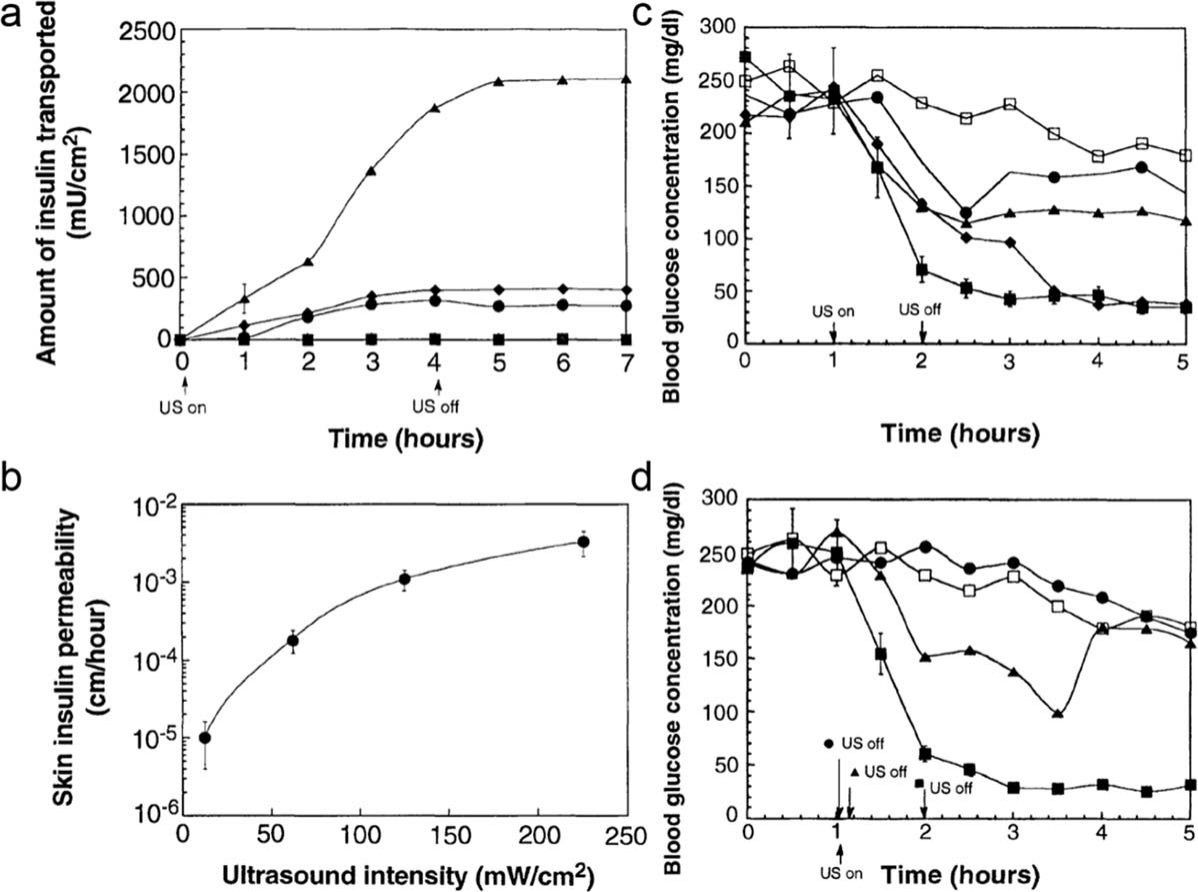
Ultrasound‐mediated transdermal insulin delivery. (a) Time variation of the amount of insulin transported across human skin (in vitro) in the presence of ultrasound (20 kHz, 100‐ms pulses applied every second) at 12.5 (▪), 62.5 (♦), 125 (•), and 225 mW/cm^2^ (▲). (b) Variation of the transdermal insulin permeability (in vitro) with ultrasound intensity (20 kHz, 100‐ms pulses applied every second). (c) Time variation of blood glucose concentrations of 16‐week‐old hairless rats on 1 hr insulin‐ultrasound treatment at 12.5 (•), 62.5 (▲), 125 (♦), and 225 mW/cm^2^ (▪). (d) Time variation of blood glucose concentration of hairless rats exposed to ultrasound (20 kHz, 225 mW/cm^2^, 100‐ms pulses applied every second) for different times. Ultrasound was turned on at 1 hr and was turned off after 1 min (•), 10 min (▲), and 1 hr (▪). Control (□). Reproduced with permission from Ref. 115

Painless transdermal drug delivery is a potential means of drug administration. However, macromolecular drugs, like proteins, are difficult to transport by this method due to the extremely low permeability of skin.^70^ Mitragotri and coworkers employed low‐frequency ultrasound to enhance the permeability to these drugs.^115^, ^116^, ^117^ They described that the application of low‐frequency ultrasound could induce growth and oscillations of air pockets present in the keratinocytes of the stratum corneum, and then disorganize the lipid bilayers to enhance transdermal delivery efficacy. With the increase in skin permeability, insulin was readily diffused into body (Figure 5a and b). Moreover, the application of ultrasound did not decrease the barrier properties of the skin after 24 hr. In vivo studies showed after transdermal delivery of insulin with ultrasound, the BGLs could significantly reduce to a normal range in rats (Figure 5c and d), and even in a large animal model‐pig.^118^, ^119^

Kwok et al. designed a polymeric monolith coated with an ultrasound‐responsive shell for insulin delivery.^120^ Relatively impermeable and ordered methylene chains formed this ultrasound‐controlled “on–off” switch by self‐assembling. In the absence of ultrasound, the orderly structure was able to keep the drug inside the polymer carrier. While with the assistance of ultrasound, the encapsulated insulin quickly diffused from the distorted crystallized self‐assembled surface structure, achieving a pulsatile drug delivery. Di et al. developed an ultrasound‐triggered insulin delivery system based on an injectable nano‐network,^121^ which was formed by the mixing and interacting of positively and negatively charged poly(lactic‐*co*‐glycolic acid) (PLGA) NPs. When a focused ultrasound system (FUS) was applied to localize acoustic energy in the small injected area of the nano‐network, the dissociation of the nano‐network may promote the insulin release. Once the ultrasound was withdrawn, the strong cohesive property allowed the nano‐network to recover and stopped further insulin release. The results on a diabetic mouse model demonstrated that the injected nano‐network could effectively be triggered to release insulin for multiple times and regulate BGLs through a portable FUS within 10 days.

### 4.2 Light‐triggered drug delivery

**Figure 6:**
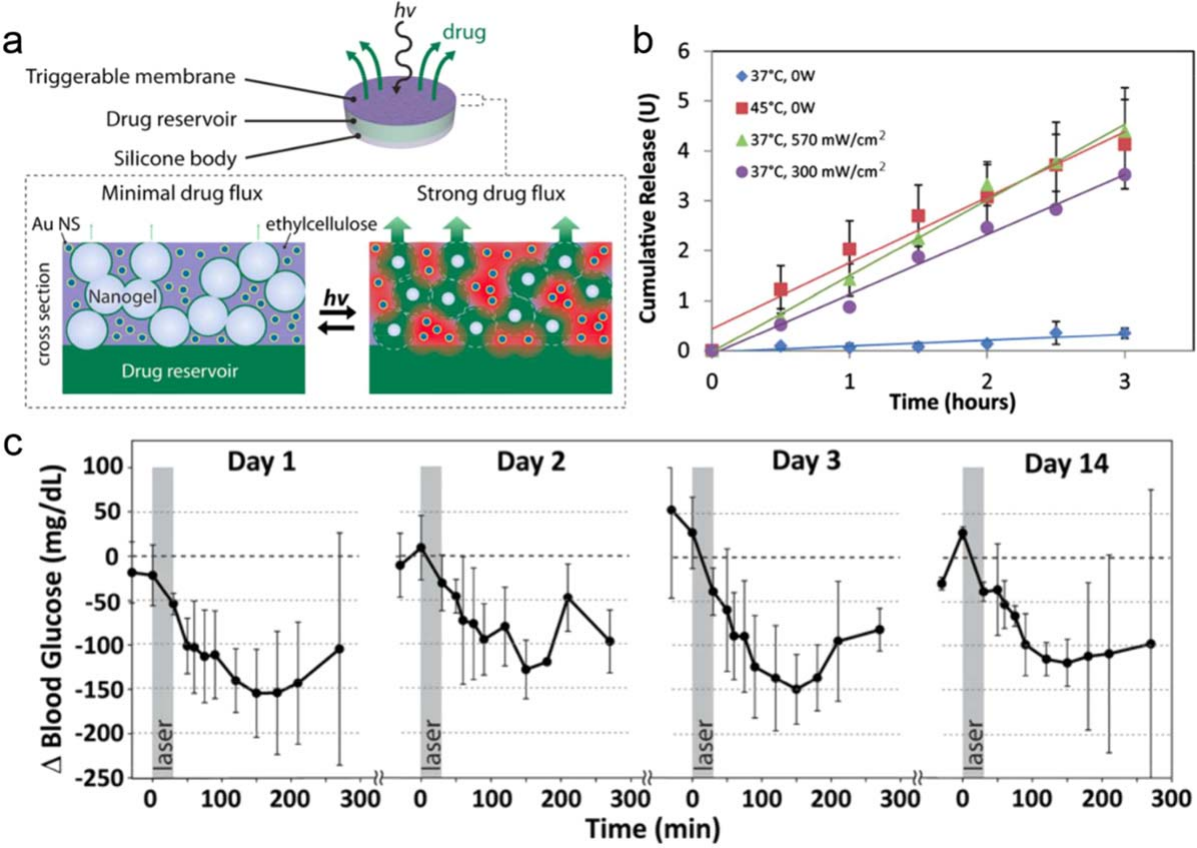
(a) Schematic of proposed device (upper) and membrane cross‐section (lower). (b) The device was turned on by immersion in a 45°C bath or laser irradiation at 570 or 300 mW/cm^2^. In all cases, the release rate was constant over at least 3 hr, and was much greater than release from the same devices in the off state (blue). (c) Blood glucose levels after repeated dosing at the same irradiance (gray box; 30 min duration; 570 mW/cm^2^) on four separate occasions over 14 days. Reproduced with permission from Ref. 123

Using near‐infrared (NIR) light irradiation, the controlled transdermal insulin delivery was achieved by Nose et al.^122^ They used a complex of gold nanorods (GNRs) and an edible surfactant to entrap insulin in an oil phase, forming a solid‐in‐oil (SO) formulation (SO‐INS‐GNR). Both surfactant hydrophobicity and the oil phase in the formulation could help SO‐INS‐GNR to penetrate through the lipid pathway of the skin. Furthermore, the GNR core as a NIR light‐responsive material was utilized to control the penetration depth under irradiation. The GNRs absorbed NIR light and then converted light energy into heat, which could break the stratum corneum of the skin (Figure 6a, b). In vivo experiments on diabetic mice demonstrated that SO‐INS‐GNR with NIR light irradiation effectively led to a long‐lasting decrease in BGLs (Figure 6c). More recently, Langer, Kohane, and coworkers also reported a NIR‐actuated device for remotely controlled insulin delivery.^123^ This device consists of an insulin reservoir and a hydrophobic ethylcellulose membrane containing gold NPs. On exposure to NIR irradiation, the gold NPs were heated, leading to the reversible collapse of the network of interconnected polymer nanoparticles. The resulting porous structure allowed insulin to rapidly diffuse. By modulation of irradiation, they created an on‐demand, reproducible, repeated, and tunable insulin dosing in diabetic rats after s.c. implantation.

### 4.3 Temperature‐triggered drug delivery

**Figure 7:**
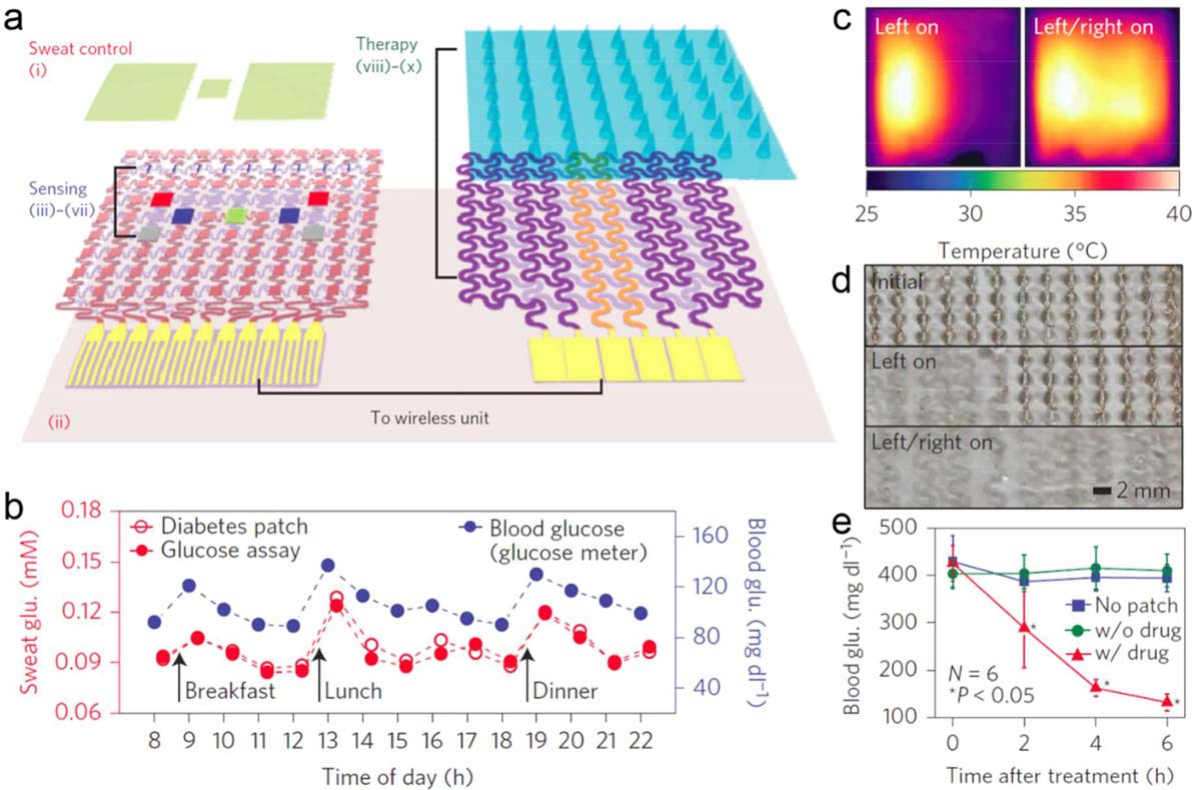
(a) Schematic drawings of the diabetes patch, which is composed of the sweat‐control, sensing, and therapy components. (b) One‐day monitoring of glucose concentrations in the sweat and blood of a human. (c) Infrared camera images of multichannel heaters showing the stepwise drug release. (d) Optical images of the stepwise dissolution of the microneedles. (e) Blood glucose concentrations of mice for the treated group (with the drug) and control groups (without the patch and without the drug). Reproduced with permission from Ref. 127

Based on thermal‐responsive polymers such as poly(NIPAM) (PNIPAAm), several temperature‐triggered insulin delivery systems have been developed.^124^, ^125^ Lima et al. prepared a photo‐crosslinked methacrylated dextran/PNIPAAm bead containing insulin.^126^ The resulting beads exhibited temperature‐dependent swelling, and the release rate of insulin was well tuned by adjusting the temperature of the medium. Integration with a grapheme‐based electrochemical sensor lead to a thermo‐responsive microneedle‐array patch, which was developed for real‐time glucose control by Kim and coworkers.^127^ The graphene doped with gold when combined with a gold mesh showed an improved electrochemical activity to satisfy the requirements for a wearable sensing and actuator patch. The integrated system was comprised of three modules: a sweat‐uptake layer, multifunctional sensors, and a Metformin (an antidiabetic drug)‐load patch with a heater (Figure 7a). The grapheme‐hybrid glucose and pH sensors could detect electrochemical signal changes by the reduction of H_2_O_2_ generated through oxidation of glucose in the sweat (Figure 7b). After comparing the real‐time glucose monitoring data with preprogrammed thresholds of the acceptable glucose concentration, the heater embedded in the Metformin (an anti‐diabetic drug)‐loaded patch was turned on wirelessly (Figure 7c). The thermally active bioresorbable coating layer of PCM was then quickly dissolved and released drug into the bloodstream (Figure 7d and e). To control the insulin dose, they designed a patterned heating to trigger drug release in a stepwise manner.

### 4.4 Other physical signals‐triggered drug delivery

Electrical potentials have also been exploited as a trigger to activate insulin release by Shi and coworkers.^128^ They fabricated a multilayered chitosan/layered double hydroxides (LDHs) hybrid hydrogel via a single electrodeposition process. Insulin was further adsorbed on the surface of the LDHs through electrostatic interactions with a high loading capability (20%). When applying a positive or negative potential, the pH of the chitosan/LDHs hydrogel environment shifted, leading to a faster insulin release. Through adjustment of the electrical signals, different release rates could be achieved under a physiological condition. They also observed that the insulin release rate was tuned by addition of different anions due to the interaction with Mg‐sites and Al‐sites. Similarly, Shamaeli et al. also developed an electrical/pH dual stimuli‐responsive nanobiocomposite for smart insulin delivery.^129^ This functionalized gold NP‐polypyrrole‐nanobiocomposite (PPy‐FGNP‐NBC) with a large effective surface area was fabricated by a simple electrodeposition and immobilization process. The insulin‐surface binding ability was strongly affected by pH and external potential stimuli. The release rate slowed down in an artificial gastric juice (pH 1.2), while it increased in an artificial intestinal fluid (pH 6.8). Furthermore, this pH‐sensitivity was remarkably enhanced by applying potential, thus displaying its potential application in oral delivery of insulin.

**Figure 8:**
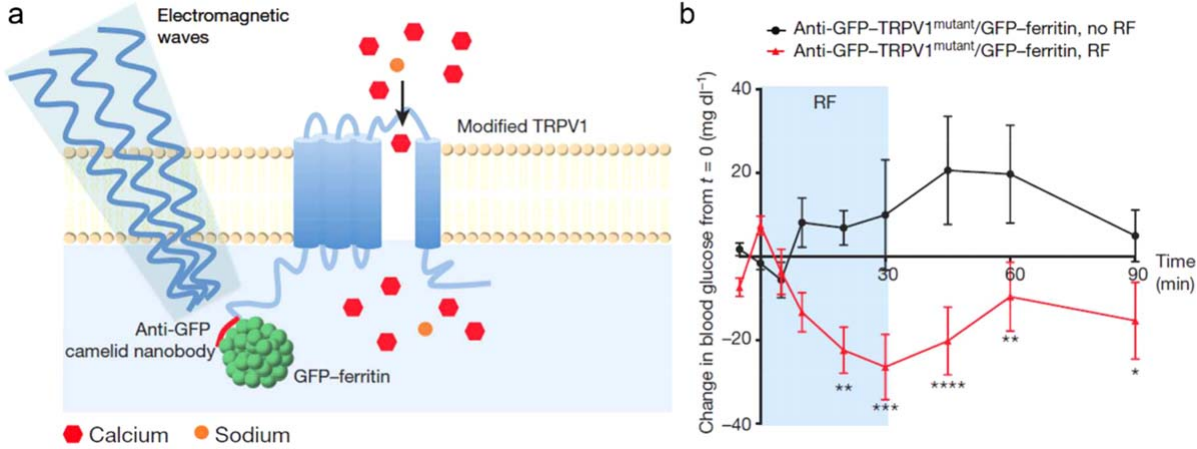
Remote neural activation in vivo using radio waves. (a) Schema of activation system. (b) Change in blood glucose with RF treatment of GK–Cre mice with ventromedial hypothalamus injection of Ad‐FLEX‐anti‐GFP‐TRPV1^mutant^/GFP‐ferritin. Reproduced with permission from Ref. 130

Friedman and coworkers applied a modified thermo‐sensitive channel, transient receptor potential cation channel subfamily V member 1 (TRPV1) for radio wave‐mediated secretion of insulin.^113^ The temperature‐sensitive TRPV1 was decorated with antibody‐coated iron oxide NPs. When exposed to radio waves, the iron oxide NPs increased the local temperature, further stimulating TRPV1 to gate calcium and subsequent release of bioengineered insulin driven by a Ca^2+^‐sensitive promoter. More recently, the same group utilized radio waves or magnetic fields to control glucose homeostasis without the exogenous ferritin NPs.^130^ The activation of a glucose‐sensing neuron was accomplished via Cre‐dependent expression of a GFP‐tagged ferritin fusion protein tethered to the cation‐conducting TRPV1 by an anti‐GFP antibody (anti‐GFP–TRPV1) (Figure 8a). A replication‐deficient adenovirus with Cre‐dependent expression of anti‐GFP–TRPV1/GFP–ferritin was injected unilaterally into the ventromedial hypothalamus of glucokinase–Cre (GK–Cre) mice. Radio frequency treatment could activate glucose‐sensing neurons to raise BGLs and glucagon levels, while inhibition reduced BGLs and increased insulin levels (Figure 8b). Besides controlling the insulin secretion, magnetic field can also be utilized to guide and extend the residence time of magnetically responsive particles to increase delivery efficacy for oral administration. Based on this concept, Langer and coworkers demonstrated the external magnetic field could localize magnetite‐containing insulin vehicles in the intestinal area, and released insulin amount was significantly increased in this condition, leading to an improved hypoglycemic effect.^131^

Recently, Gu and coworkers designed a stretch‐triggered insulin delivery system using wearable elastomer films containing therapeutic depots.^132^ The microgel depots consisting of insulin loaded NPs were embedded about halfway inside the elastomeric film. The encapsulated insulin in the NPs was passively released and partially stored in the matrix of the microdepots. On stretching, the microdepot followed the deformation of the elastomeric substrate, which facilitated the drug release rate due to the enlarged surface area for diffusion and Poisson's ratio‐induced compression on the microdepot. Through integration with a painless microneedle‐array patch, this wearable drug delivery device can be utilized for transcutaneous drug delivery. They demonstrated that the BGLs of the mice wearing this device declined rapidly to a normal state within 30 min after applying 10 cycles of stretching with a strain level of 50% by hand. This stretching‐triggered MN‐based system provides a new strategy for on‐demand glycemia control in a convenient manner.

## 5 Conclusions

Stimuli‐responsive anti‐diabetic drug delivery systems have exhibited tremendous therapeutic potency for diabetes treatment. Unlike traditional subcutaneous injections, which often rely on a historical understanding of the patient's BGL profile in response to daily meals and anti‐diabetic medication to determine insulin dosages,^133^ the smart drug delivery systems with stimuli‐trigger mechanisms, especially glucose‐responsive based closed‐loop systems, have the potential to improve the compliance and quality of life for diabetic patients. In addition, utilizing physiological factors as the trigger, a sustained release of therapeutics can be achieved for long‐term diabetes treatment. For example, Chilkoti and coworkers reported an injectable protease operated depot (POD) for GLP‐1 delivery.^134^ In this study, GLP‐1 was fused with a thermally sensitive elastin‐like polypeptide through an arginine‐glycine protease cleavage site. The POD released GLP‐1 by protease cleavage between copies of the peptide, allowing prolonged release of bioactive drug into circulation. Peppas and coworkers developed an insulin‐loaded hydrogel crosslinked by biodegradable oligopeptide.^135^ The trypsin in the small intestine could degrade the microgel, leading to a sustained insulin release.

In spite of the advancements illustrated in this review, there are still more efforts needed for potential clinical applications. First, how to achieve fast responsiveness in vivo is a highly urgent problem that needs to be solved. Currently, many formulations have shown ideal on‐demand fast action in in vitro experiments.^13^, ^45^ However, their in vivo performances are still unsatisfactory due to the complicated physiological environment. Second, the doses and release rates of insulin are supposed to be precisely quantified. The burst release of insulin from depot‐based systems may result in hypoglycemia, which may cause a variety of symptoms including behavioral and cognitive disturbance, seizure, loss of consciousness, brain damage, and even death.^136^ A combination delivery system^137^ that can simultaneously release glucagon in response to low BGLs is envisioned to avoid risk of hypoglycemia. Third, it is important to achieve real‐time glucose monitoring for external remote triggered system. A non‐invasive glucose sensor may act as an ideal activator to control the external signals to promote drug release. Last but not least, biocompatibility and non‐toxicity of stimuli‐responsive formulations should be carefully assessed. Since diabetes treatment is a long‐term, even lifetime, administration any possible side effects may lead to serious problems.^138^
